# *Zmat2* in mammals: conservation and diversification among genes and Pseudogenes

**DOI:** 10.1186/s12864-020-6506-3

**Published:** 2020-01-31

**Authors:** Peter Rotwein, Kabita Baral

**Affiliations:** 1 Department of Molecular and Translational Medicine, Paul L. Foster School of Medicine, Texas Tech Health University Health Sciences Center, El Paso, TX 79905 USA; 2 Graduate School, College of Science, University of Texas at El Paso, El Paso, TX 79902 USA

**Keywords:** ZMAT2, Gene structure, Gene evolution, Database analysis

## Abstract

**Background:**

Recent advances in genetics and genomics present unique opportunities for enhancing our understanding of mammalian biology and evolution through detailed multi-species comparative analysis of gene organization and expression. Yet, of the more than 20,000 protein coding genes found in mammalian genomes, fewer than 10% have been examined in any detail. Here we elucidate the power of data available in publicly-accessible genomic and genetic resources by querying them to evaluate *Zmat2*, a minimally studied gene whose human ortholog has been implicated in spliceosome function and in keratinocyte differentiation.

**Results:**

We find extensive conservation in coding regions and overall structure of *Zmat2* in 18 mammals representing 13 orders and spanning ~ 165 million years of evolutionary development, and in their encoded proteins. We identify a tandem duplication in the *Zmat2* gene and locus in opossum, but not in other monotremes, marsupials, or other mammals, indicating that this event occurred subsequent to the divergence of these species from one another. We also define a collection of *Zmat2* pseudogenes in half of the mammals studied, and suggest based on phylogenetic analysis that they each arose independently in the recent evolutionary past.

**Conclusions:**

Mammalian *Zmat2* genes and ZMAT2 proteins illustrate conservation of structure and sequence, along with the development and diversification of pseudogenes in a large fraction of species. Collectively, these observations also illustrate how the focused identification and interpretation of data found in public genomic and gene expression resources can be leveraged to reveal new insights of potentially high biological significance.

## Background

Of the more than 20,000 protein coding genes found in human and in other mammalian genomes, fewer than 10% have been studied in any detail [1–3]. This is true despite that fact that ready access to public genomic and gene-expression databases [4] means that nearly any gene is available for intensive analysis from the molecular and cellular to the individual and population levels [5–10]. Part of this disparity may reflect social or historical reasons, but it also is likely that direct association with human diseases and the ready availability of experimental models influences decisions to gravitate toward scientific areas that appear more amenable to higher profile publications or grant funding [2, 3].

*ZMAT2* is an excellent example of a gene that had essentially been unstudied until late 2018 [11]. *ZMAT2*, which encodes a protein that contains a zinc finger domain, is part of a 5-gene family of limited intra-familial amino acid similarity except for the zinc finger region. The lack of interest in this gene is potentially surprising, since it is the ortholog of Snu23, a yeast protein that plays an important role in the spliceosome [12], an essential molecular machine in eukaryotes that removes introns from primary gene transcripts [13]. Although human ZMAT2 also has been mapped to the spliceosome in structural biological studies [14], even this observation has not much generated interest in the protein.

Here, by using information extracted from public repositories, we have studied *Zmat2* genes and proteins from a broad group of 18 mammalian species comprising 13 orders, and representing ~ 165 million years (Myr) of evolutionary diversification [15–18]. Our results show extensive conservation in coding regions of these genes and in their encoded proteins, define a collection of *Zmat2* pseudogenes in half of the mammals studied, and identify one mammal in which *Zmat2* has undergone a tandem duplication. Our observations provide an illustration of how the focused application and analysis of data found in publicly-available genomic and gene expression resources can be leveraged to reveal new insights of potentially high biological significance.

## Results

### Mammalian *ZMAT2*/*Zmat2* genes are poorly annotated in genomic databases

**Fig. 1:**
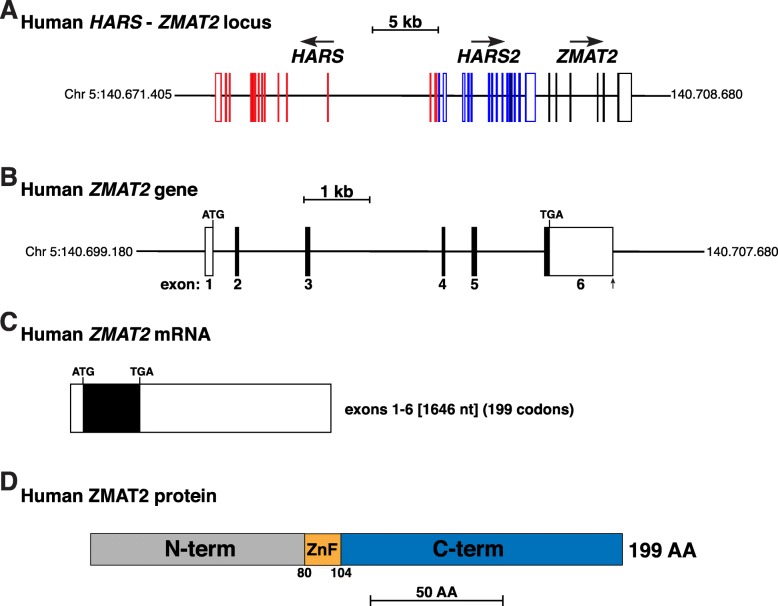
Organization of the human *ZMAT2* locus and gene. **a**. Diagram of the human *HARS*-*HARS2*-*ZMAT2* locus on chromosome 5. Exons are depicted by lines and boxes (red for *HARS*, blue for *HARS2*, black for *ZMAT2*), with coding regions solid and non-coding regions white. The direction of transcription for each gene is indicated and a scale bar is shown. **b**. Map of the human *ZMAT2* gene. Coding regions are in black and noncoding segments in white. A scale bar is shown. **c**. Diagrams of human *ZMAT2* mRNA, as characterized in (Baral K, Rotwein P: The story of ZMAT2: a highly conserved and understudied human gene, manuscript submitted). Coding regions are labeled in black and non-coding segments in white. The length is indicated in nucleotides (nt) as are the number of codons in the open reading frame. **d**. Schematic of human 199-residue ZMAT2 protein, with NH_2_ (N) and COOH (C) terminal (term), and zinc finger (ZnF) regions labeled and color-coded

Human ZMAT2 is an ortholog of yeast Snu23, a zinc-finger-containing protein that is a key component of the spliceosome [12], the molecular machine responsible for the removal of introns from primary gene transcripts [13]. The human *ZMAT2* gene has been incompletely characterized in the Ensembl and UCSC genomic repositories. We thus mapped the gene and its transcripts and protein (Fig. 1, Baral K, Rotwein P: The story of ZMAT2: a highly conserved and understudied human gene, manuscript submitted). Based on these results, which also revealed that 6-exon human *ZMAT2* and its encoded 199-residue protein was highly conserved among non-human primates (Baral K, Rotwein P: The story of ZMAT2: a highly conserved and understudied human gene, manuscript submitted), we now sought to extend knowledge about *Zmat2* by defining it in other mammalian species.

A preliminary examination within Ensembl revealed that the assignments of mammalian *Zmat2* genes were even more incomplete than was observed for human *ZMAT2*, not only for the 18 species chosen here to cover a range of mammalian orders, but also for most of the mammalian and non-mammalian vertebrates in which *Zmat2* has been identified in their genomes in Ensembl. For example, 5′ untranslated regions (UTRs) in exon 1 were described in only 6 of 18 species, and 3′ UTRs in exon 6 in only 7 of 18 species (Table 1). We thus developed an iterative strategy to define these genes, in which mouse *Zmat2* was initially characterized in detail. Its exons then were used to perform homology searches in other mammalian genomes. As needed, these queries were supplemented by individual comparisons with *Zmat2* cDNAs when available in the National Center for Biotechnology Information (NCBI) nucleotide database (cDNAs were listed in this resource for only 6 different species; see Methods), and by secondary searches using *Zmat2* gene segments from species that were evolutionarily more similar to specific target species (e.g., using koala exon 1 to identify opossum exon 1). Most importantly, a final series of studies used the resources of the NCBI Sequence Read Archive (SRA) to map the putative 5′ and 3′ ends of each gene by analysis of expressed transcripts [19, 20]. As described below, results revealed substantially higher levels of gene complexity and completeness than had been found in the data curated by Ensembl.

**Table 1:** Mammalian *Zmat2* Genes in Ensembl Genome Browser

Species	Exon 1 5′ UTR (nt)	Exon 1 coding (nt)	Exon 6 coding (nt)	Exon 6 3’UTR (nt)
mouse	66	18	144	1644
rat	117	18	144	1235
guinea pig	232	18	144	2998
rabbit	None	18	144	180
cow	6	18	144	903
horse	None	18	144	None#
pig	18	18	144	1038
sheep	None	18	144	None^
goat	104	18	144	None
dog	None	18	144	None
cat	None	18	75	None
elephant	None	18	80	None
dolphin	None	18	144	None
microbat	None	18	144	None
megabat	None	18	144	None
opossum	None	18	144	2630
Tas. devil	None	18	144	None
koala	None	18	144	None*

#691 base pairs are found in *Zmat2* cDNA JL616468 in NCBI

nucleotide database

^nucleotide database

*922 base pairs are found in *Zmat2* cDNA XM_021005188 in NCBI

nucleotide database

### The mouse *Zmat2* gene

**Fig. 2:**
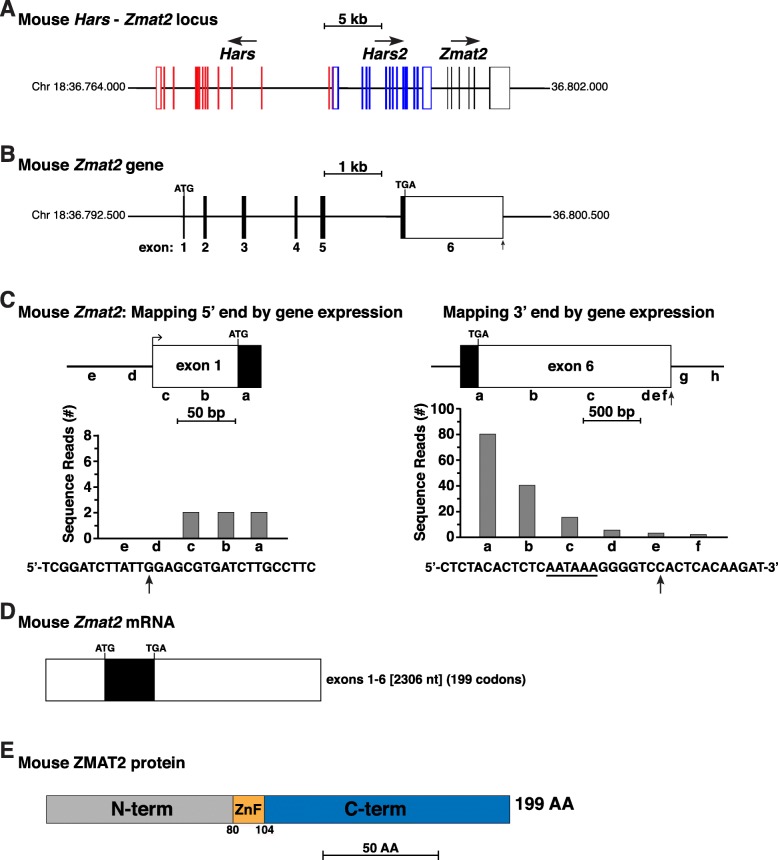
Characterization of the mouse *Zmat2* locus and gene. **a**. Schematic of the mouse *Hars*-*Hars2*- *Zmat2* locus on chromosome 18. Exons are shown as lines and boxes (red for *Hars*, blue for *Hars2*, black for *Zmat2*); coding regions are solid and non-coding segments white. The direction of transcription is indicated for each gene and a scale bar is shown. **b**. Map of the mouse *Zmat2* gene as depicted in the Ensembl genome database. Coding regions are in black and noncoding segments in white. A scale bar is shown. **c**. Mapping the beginning and end of mouse *Zmat2*: diagram of mouse *Zmat2* exon 1 (left) and exon 6 (right), and graphs of gene expression data from the SRA NCBI RNA-sequencing library, SRX116916 (Additional file 1: Table S1), using 60 base pair genomic segments a-e, and a-f, respectively, as probes. The DNA sequence below the left graph depicts the putative 5′ end of exon 1, with the locations of the 5′ end of the longest RNA-sequencing clone indicated by a vertical arrow. Shown below the right graph is the DNA sequence of the putative 3′ end of exon 6. A potential polyadenylation signal (AATAAA) is underlined and a vertical arrow denotes the possible 3′ end of *Zmat2* transcripts. **d**. Diagram of the mouse *Zmat2* mRNA. Coding regions are in black and non-coding segments in white. The length is indicated in nucleotides (nt), as are the number of codons in the open reading frame. **e**. Schematic of the mouse ZMAT2 protein, with NH_2_ (N) and COOH (C) terminal (term), and zinc finger (ZnF) regions labeled and color-coded

A search of Ensembl revealed that mouse *Zmat2* appeared to be a 6-exon gene on chromosome 18, and like human *ZMAT2* was located adjacent to *Hars2* in the same transcriptional orientation (compare Fig. 2a and Fig. 1a). Of two proposed mouse *Zmat2* transcripts in Ensembl, only one was stated to include all 6 exons (Fig. 2b) and to encode a protein of 199-amino acids, while the other was thought to include parts of 3 exons and a retained intron (see: https://useast.ensembl.org/Mus_musculus/Gene/Summary?db=core;g=ENSMUSG00000001383;r=18:36793876-36799666). Inspection of the presumptive full-length *Zmat2* transcript revealed a proposed 5′ UTR of 66 base pairs (Table 1), that could not be extended by comparison with *Zmat2* cDNA NM_025594 from the NCBI nucleotide database (5′ UTR of 19 base pairs).

Direct analysis of mouse *Zmat2* gene expression using RNA-sequencing libraries from liver and keratinocytes (Additional file 1: Table S1) revealed that transcripts containing *Zmat2* exon 1 were expressed at low levels (read counts of no more than 2 sequences per probe, Fig. 2c). Nevertheless, examination of these libraries revealed that exon 1 was at least 96 nucleotides in length (Fig. 2c). However, no potential TATA boxes, which position RNA polymerase II at the start of transcription [21], or initiator elements, which function similarly [22], were found adjacent to this transcript. Thus, the 5′ end of the mouse *Zmat2* gene remains tentatively mapped.

Similar studies using probes from different parts of exon 6 showed that this exon was 1774 nucleotides in length, and thus was ~ 14 nucleotides shorter than stated in Ensembl. The 3′ end of exon 6 contained an ‘AATAAA’ presumptive poly A recognition sequence, and a poly A addition site [23] was mapped 7 base pairs further 3′ (Fig. 2c), thus supporting our analysis. Taken together, these results describe a 6-exon mouse *Zmat2* gene of 5786 base pairs in length (Table 2), that is transcribed and processed into a mRNA of 2306 nucleotides (Fig. 2d), and that encodes a 199-amino acid ZMAT2 (Fig. 2e).

**Table 2:** Characterization of Mammalian *ZMAT2* Genes (in base pairs)

Species	Exon 1	Intron 1	Exon 2	Intron 2	Exon 3	Intron 3	Exon 4	Intron 4	Exon 5	Intron 5	Exon 6	Total Length
mouse	96	337	94	650	124	872	74	388	146	1234	1774	5786
rat	35	331	94	653	124	1084	74	409	146	1029	1847	5826
guinea pig	125	351	94	1391	124	2670	74	1008	146	1042	1064	8089
rabbit	120	332	94	1451	124	1517	74	1784	146	2061	3263	10,966
cow	300	332	94	939	124	1201	74	419	146	696	2688	7013
horse	225	335	94	1066	124	1758	74	450	146	781	2899	7952
pig	114	332	94	1103	124	926	74	700	146	1352	1044	6009
sheep	60	333	94	940	124	1199	74	432	146	908	1031	5341
goat	134	356	94	940	124	1200	74	432	146	988	1046	5534
dog	281	335	94	1063	124	2801	74	462	146	696	> 4381	> 10,457
cat	245	336	94	1077	124	1427	74	440	146	718	> 4105	> 8786
elephant	180	332	94	1076	124	1447	74	440	146	717	1066	5696
dolphin	175	326	94	1217	124	1244	74	751	146	834	1212	6197
microbat	#18	296	94	1686	124	1679	74	783	146	1141	#144	6185
megabat	89	333	94	945	124	1237	74	713	146	680	1042	5477
opossum 1	*467	732	94	1689	124	661	74	609	146	1595	2751	8942
opossum 2	*467	723	94	1792	124	661	74	608	146	1498	2751	8938
Tas. devil	146	709	94	2946	124	659	74	670	146	616	2004	8188
koala	239	648	94	2094	124	684	74	660	146	861	2311	7935

#No RNA-sequencing libraries express *ZMAT2*

*The 5′ ends of these genes converge (see Fig. 4)

### The *Zmat2* gene in other mammals

**Fig. 3:**
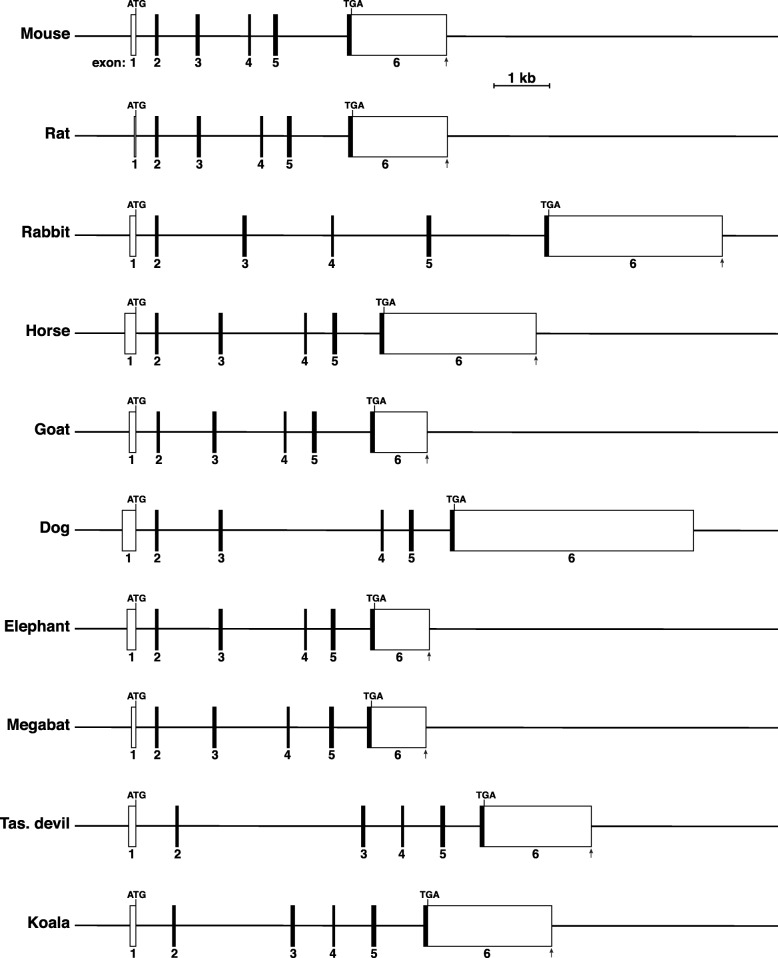
The *Zmat2* gene in mammals. Diagrams of mouse, rat, rabbit, horse, goat, dog, elephant, megabat, Tasmanian (Tas.) devil, and koala *Zmat2* genes. For each gene, exons are indicated as boxes, with coding regions in black and non-coding segments in white. A scale bar is shown. See Tables 2 and 3 for more details

By searching genome databases with mouse exons, the few homologous cDNAs, and in selected cases, exons from closely related species, *Zmat2* was characterized in 17 other mammals representing 9 different orders, and spanning ~ 165 Myr of evolutionary history. These other mammalian *Zmat2* genes also all appeared to consist of 6 exons (Fig. 3, Table 2), and when their 5′ and 3′ ends were mapped using species-homologous RNA-sequencing libraries (Additional file 1: Table S1, Additional file 2: Table S2, Additional file 3: Figure S1, Additional file 4: Figure S2 and Additional file 5: Figure S3), their overall structures closely resembled mouse *Zmat2* (Fig. 3, Table 2). In particular, there was perfect congruence in the lengths of coding exons 2–5 (Table 2), and high levels of DNA sequence identity (84.3 to 97.8%, Table 3). Total gene sizes varied over a 2-fold range, from 5477 base pairs in megabat to > 10,457 base pairs in dog, with most of the differences attributable to longer or shorter 3′ UTRs in exon 6 and to some variation in intron lengths (Table 2).

**Table 3:** Nucleotide Identity with Mouse *ZMAT2* Exons

Species	Exon 1 (96 bp)*	Exon 2 (94 bp)	Exon 3 (124 bp)	Exon 4 (74 bp)	Exon 5 (146 bp)	Exon 6 (1774 bp)*
rat	96.8	97.8	94.3	97.3	96.6	87.5
guinea pig	89.3	92.3	87.7	94.0	90.4	78.8 (816 bp)
rabbit	91.7	91.5	87.7	88.1	93.8	80.2 (960 bp)
cow	88.6	94.7	88.5	98.6	90.4	84.8 (887 bp)
horse	91.7	94.7	91.8	95.7	92.5	81.3 (941 bp)
pig	90.0	95.7	89.7	94.3	89.7	86.5 (788 bp)
sheep	88.6	94.7	87.7	97.3	90.4	85.1 (781 bp)
goat	88.6	94.7	87.7	91.4	90.4	85.1 (781 bp)
dog	95.6	94.7	86.1	94.3	90.4	87.8 (886 bp)
cat	86.5	93.4	84.3	94.3	90.3	79.6 (887 bp)
elephant	95.4	94.5	89.3	94.3	91.1	79.8 (887 bp)
dolphin	87.1	94.7	88.5	95.9	92.5	84.2 (887 bp)
microbat	97.1	94.7	91.0	94.3	89.7	84.0 (887 bp)
megabat	93.3	94.7	85.3	94.3	90.4	88.1 (887 bp)
opossum 1	56.8	94.3	91.0	92.9	85.7	79.9 (163 bp)
opossum 2	55.8	94.3	91.0	92.9	84.9	78.5 (163 bp)
Tas. devil	96.7 (31 bp)^#^	96.9	90.2	90.0	85.2	87.7 (138 bp)
koala	94.3 (35 bp)	91.5	89.3	91.4	85.9	89.1 (138 bp)
human	80.8	92.6	87.7	94.3	93.2	86.7 (699 bp)

*coding and non-coding DNA

^#^Information in brackets delineates the extent of DNA similarity for exons 1 and 6

DNA conservation also was relatively high for *Zmat2* exon 1 among the mammals studied (87.1 to 96.8% identity, Table 3), even though it is comprised primarily of 5′ UTR. The exception here is opossum (55.8 and 56.8% identity, Table 3 and see below). Exon 6 was more dissimilar among the different species (Table 3), particularly in the noncoding segments (e.g., no identity in Tasmanian devil or koala).

### The opossum genome contains tandem *Zmat2* genes

**Fig. 4:**
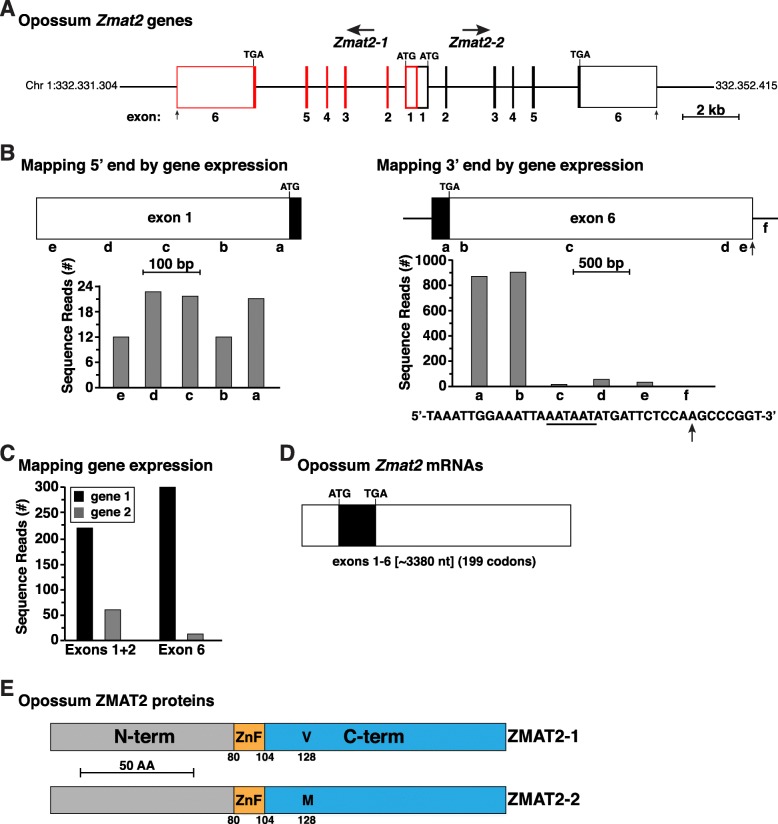
Tandem *Zmat2* genes in the opossum genome. **a**. Diagram of the opossum *Zmat2* locus on chromosome 1, showing two *Zmat2* genes, termed here *Zmat2–1* and *Zmat2–2*, and their divergent transcriptional orientations. Exons are depicted as boxes, red for *Zmat2–1* and black for *Zmat2–2*, with coding segments solid, and noncoding regions white. **b**. Mapping the beginning and end of opossum *Zmat2–1 and 2–2*: diagram of exon 1 (left) and exon 6 (right), and graphs of gene expression data from the SRA NCBI RNA-sequencing library, SRX3040092 (Additional file 1: Table S1), using 60 base pair genomic segments a-e, and a-f, respectively, as probes. Shown below the right graph is the DNA sequence of the putative 3′ end of exon 6. A potential polyadenylation signal (AATAAT) is underlined and a vertical arrow denotes the possible 3′ end of *Zmat2* transcripts. **c**. Gene expression data from SRX3040092 for each opossum *Zmat2* gene, using probes for exons 1 + 2, and exon 6 (Additional file 2: Table S2) that discriminate between transcripts from *Zmat2–1* and *Zmat2–2*. **d**. Diagram of opossum *Zmat2* transcripts. Both genes produce mRNAs that are identical in length, and are 99.9% identical in DNA sequence. The coding segment is in black and non-coding regions are in white. The length is in nucleotides and the number of codons in the open reading frame are listed. **e**. Diagram of opossum ZMAT2 proteins, with NH_2_ (N) and COOH (C) terminal (term), and zinc finger (ZnF) regions labeled and color-coded. The signal amino acid substitution at position 128 is labeled (V in ZMAT2–1, and M in ZMAT2–2

Initial screening of the opossum genome revealed several sets of DNA sequences with comparable levels of identity with mouse *Zmat2* exons 2–5 (84.9 to 94.3%, Table 3). Two of these groups of DNA segments were distributed to adjacent locations in the opossum genome, and when compiled and evaluated in detail (including identifying exon 1 by using koala *Zmat2* exon 1) consisted of tandem genes that were oriented ‘head-to-head’ in divergent transcriptional direction (Fig. 4a). Further analysis showed that the 5′ ends of exon 1 of both genes potentially overlapped (Fig. 4a, b), that exons 1 through 5 were 99.73% identical, that the lengths of exon 6 matched each other and that they were 99.9% identical in DNA sequence (Fig. 4b and not shown). By using probes that differed by a single nucleotide (Additional file 2: Table S2) to screen an RNA-sequencing library, we found that both opossum *Zmat2* genes were expressed, at least in liver, with transcripts for gene 1 being more abundant than those for gene 2 (Fig. 4c). Moreover, both opossum *Zmat2* mRNAs were the same length (Fig. 4d), and they encoded proteins that varied by a single amino acid (valine at position 128 in protein 1, and methionine in protein 2 (Fig. 4e).

### Multiple *Zmat2* pseudogenes arose independently in different mammalian genomes

**Fig. 5:**
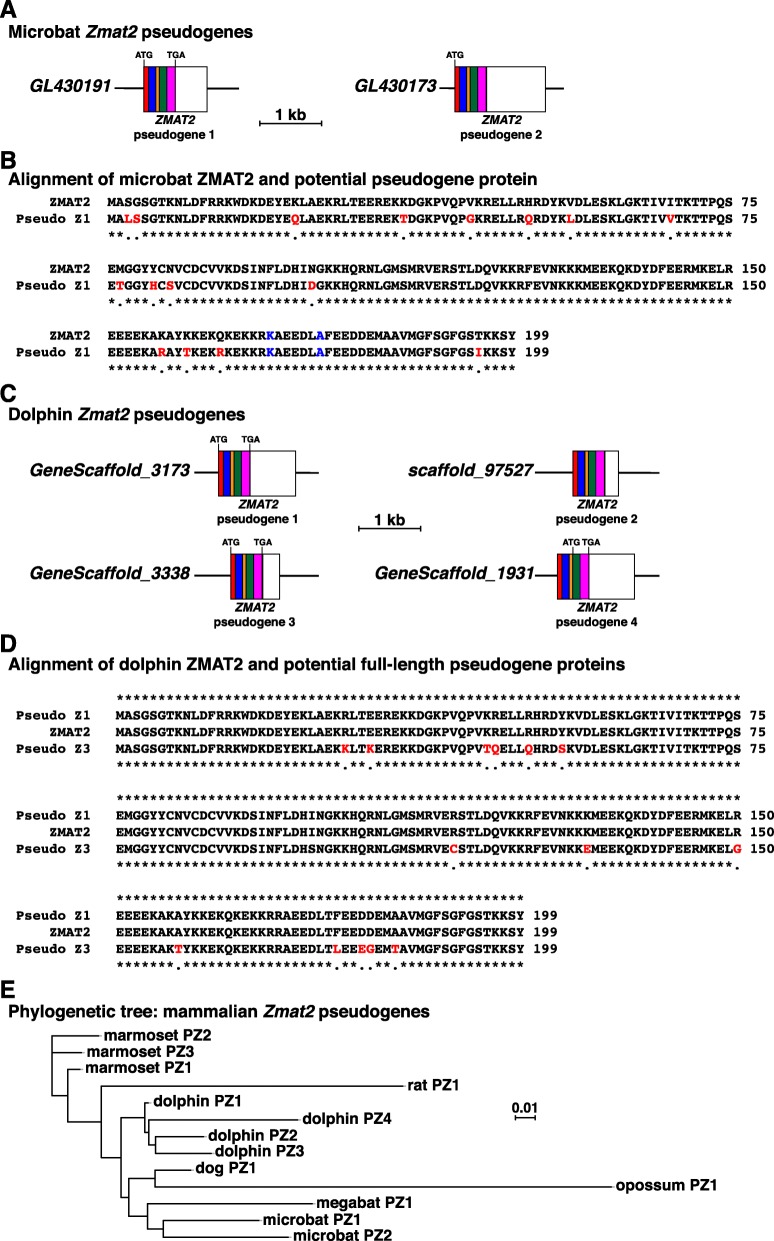
Mammalian genomes contain multiple *Zmat2* pseudogenes. **a**. Schematic of the two *Zmat2* pseudogenes in the microbat genome. The color-coding indicates regions of each pseudogene that are similar in DNA sequence to individual coding segments of authentic *Zmat2* (red – exon 2; blue – exon 3; yellow – exon 4; green – exon 5; pink – coding region of exon 6). The white areas depict segments similar to the 3′ UTR of authentic *Zmat2* exon 6 in each pseudogene. A scale bar is shown. **b**. Alignment of amino acid sequences of microbat ZMAT2 and the predicted pseudogene protein (Z1). Similarities and differences are shown, with identities being indicated by asterisks. Differences are marked in red text. The blue text denotes the two amino acids that are different from mouse or human ZMAT2 (also see Fig. 7). **c**. Schematic of the four *Zmat2* pseudogenes in the dolphin genome. The color-coding indicates regions of each pseudogene that are similar in DNA sequence to individual exons of authentic *Zmat2*, as per part **a** above, and the white areas depict segments similar to the 3′ UTR of authentic *Zmat2* exon 6 in each pseudogene. A scale bar is shown. **d**. Alignment of amino acid sequences of dolphin ZMAT2 and predicted pseudogene proteins (Z1 and Z3). Similarities and differences are shown, with identities being indicated by asterisks. Differences also are marked in red text. **e**. Phylogenetic tree of mammalian *Zmat2* pseudogenes. The data on marmoset are from (Baral K, Rotwein P: The story of ZMAT2: a highly conserved and understudied human gene, manuscript submitted). The scale bar indicates 0.01 substitutions per site and the length of each branch approximates the evolutionary distance

Screening of different mammalian genomes with individual mouse *Zmat2* exons led to the identification of additional related DNA sequences in nine species (rat, guinea pig, rabbit, dog, dolphin, microbat, megabat, opossum, and platypus; Table 4). The levels of similarity with mouse *Zmat2* exons ranged from 80.1 to 93.4% identity (Table 4). In rat, rabbit, dog, dolphin, megabat, microbat, and opossum paralogs of all 6 *Zmat2* exons were detected, and except for rabbit, were composed of continuous DNA sequences (Table 4, Fig. 5). In the latter an unreadable DNA segment of ~ 406 nucleotides separated ‘exons’ 2 and 3. These ‘full-length’ DNAs thus appeared to be pseudogenes that resembled processed mRNAs, and that presumably were retro-transposed as DNA copies back into the respective genomes [24]. In guinea pig, paralogs of only ‘exons’ 4 through 6 could be found, in platypus, individual representations of ‘exon 2’ and ‘exon 3’ mapped to different locations in the genome, and in rat two copies of 461 base pairs of ‘exon 6’ were found in different parts of the X chromosome (87.4% identity with the corresponding portions of the mouse exon, Table 4). The two putative *Zmat2* pseudogenes found in the microbat genome and the four located in the dolphin genome are depicted in Fig. 5. In microbat, one of these DNA sequences contained a continuous open reading frame of 199 codons, and its conceptual translation revealed marked similarity with the microbat ZMAT2 protein (183/199 identical residues, Fig. 5b). In dolphin, in which two of the four pseudogenes encoded 199-codon open reading frames (Fig. 5c), one was predicted to be identical to authentic ZMAT2, while the other matched it in 185/199 residues (Fig. 5d).

**Table 4:** *Zmat2* pseudogenes

Species	Pseudogenes	Exons present	Nucleotide identity with mouse *Zmat2* (%)	ZMAT2 ORF	Amino acid identity with authentic ZMAT2 (%)
rat	3	1–6, 6, 6	88.4, 87.4, 87.4	none	–
guinea pig	1	4–6	80.1	none	–
rabbit	1	*1–6	87.7	none	–
dog	1	1–6	88.3	123 AA	99.2
dolphin	4	1–6	93.4, 83.8, 89.8, 86.2	199 AA, 199 AA, 90 AA, none	100, 93.0, 83.3
megabat	1	1–6	87.1	none	–
microbat	2	1–6	89.5, 81.5	199 AA, none	91.0
opossum	1	1–6	86.6	96 AA	77.1
platypus	1	#2, 3	89.8, 87.7	none	–
marmoset	3	1–6, 1–6, **1–6	91.8, 89.2, 90.9	123 AA, 199 AA, none	97.6, 98.5

*Unreadable sequence of ~ 406 base pairs separates exons 2 and 3

#Located on different contigs in genome

**Alu element separates exons 3 and 4

**Fig. 6:**
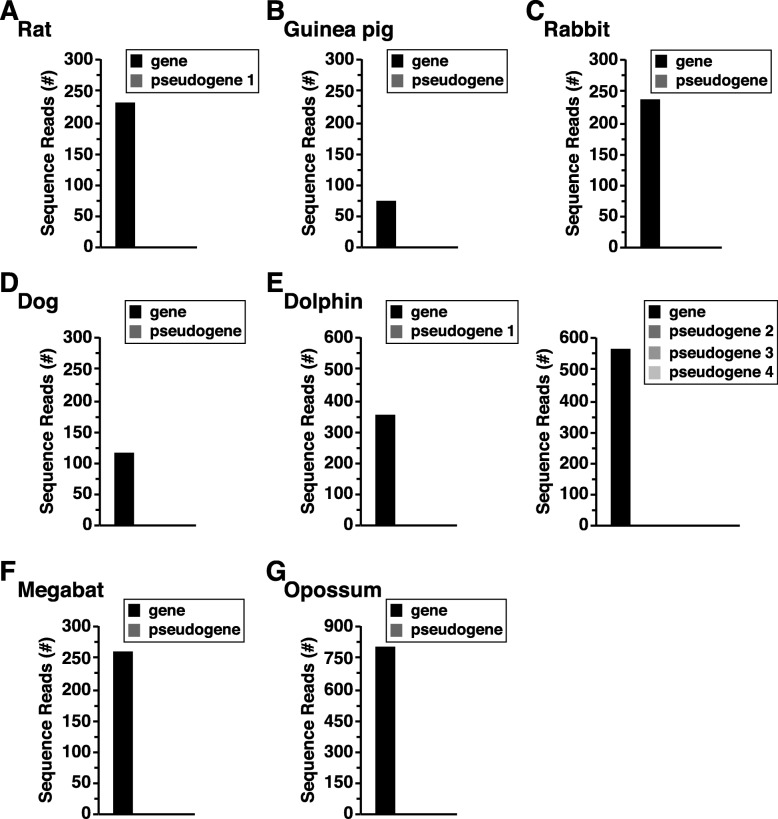
Lack of expression of mammalian *Zmat2* pseudogenes using analysis of RNA-sequencing libraries. Gene expression data were obtained by querying species-specific RNA-sequencing libraries with DNA probes that detect differences between mammalian *Zmat2* genes and potential pseudogenes. See Additional file 1: Table S1 for the list of RNA-sequencing libraries and Additional file 2: Table S2 for the DNA probes. **a**. Rat; **b**. Guinea pig; **c**. Rabbit; **d**. Dog; **e**. Dolphin; **f**. Megabat; **g**. Opossum

Previous studies have shown that some potential pseudogenes for the human protein phosphatase 1 regulatory subunit (*PP1R2*) are transcribed and thus are not actually pseudogenes since they are expressed as RNAs [25]. To determine whether or not any mammalian *Zmat2* pseudogenes are functional, their gene expression was examined by querying RNA-sequencing libraries. As shown for rat, rabbit, guinea pig, dog, dolphin, megabat, and opossum, no transcripts could be detected in these libraries even though in all cases authentic *Zmat2* mRNA was readily expressed (Fig. 6a-g; no microbat RNA sequencing library was available in the NCBI SRA).

Phylogenetic analysis of all 13 ‘full-length’ *Zmat2* pseudogenes from 7 different mammals (including marmoset (Baral K, Rotwein P: The story of ZMAT2: a highly conserved and understudied human gene, manuscript submitted), Table 4) demonstrated that the DNA sequence of each pseudogene was more closely related to the paralog or paralogs from the homologous species than to other *Zmat2* pseudogenes (Fig. 5e), suggesting that these retro-transposition events each arose independently after the divergence of each species from their nearest mammalian ancestors.

### ZMAT2 protein sequences are highly-conserved among mammals

**Fig. 7:**
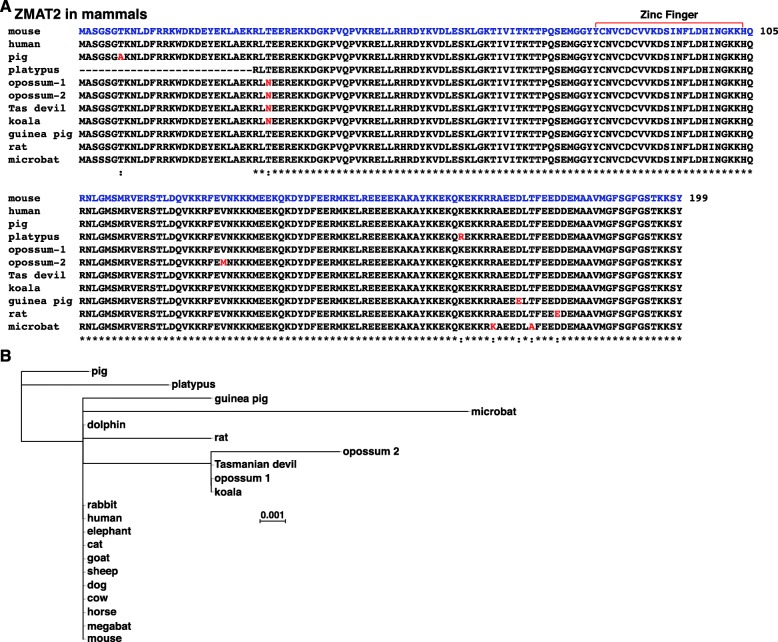
Mammalian ZMAT2 proteins. **a**. Alignments of amino acid sequences of ZMAT2 proteins from selected mammalian species are shown in single letter code. Identities and differences among species are indicated, with identities labeled by asterisks. Dashes indicating no residue have been placed to maximize alignments. The red text depicts differences from the mouse protein. The zinc finger region in highlighted. **b**. Phylogenetic tree of ZMAT2 in mammals. The protein sequences not shown in **a** are identical to mouse ZMAT2, as can be seen in the tree. The scale bar indicates 0.01 substitutions per site and the length of each branch approximates the evolutionary distance

ZMAT2 was identical to the mouse and human protein in ten species studied here (Table 5, Fig. 7a, b). In each of the other 8 species, only one or two amino acid substitutions was found, except for platypus, in which the NH_2_-terminus of the protein could not be established because of incomplete genomic sequence (Fig. 7). Phylogenetic mapping further showed that marsupial ZMAT2 proteins clustered together, as all were identical except for opossum 2 (Fig. 7b). Of note for all variant ZMAT2 proteins, the altered amino acids were located throughout the protein, but none were found in the zinc finger domain (Fig. 7a).

**Table 5:** Amino Acid Identities with Mouse ZMAT2

Species	Length	Percent Identity	Amino Acid Differences
rat	199	99.5	D^180^ > E
guinea pig	199	99.5	D^174^ > E
rabbit	199	100	–
cow	199	100	–
horse	199	100	–
pig	199	99.5	T^7^ > A
sheep	199	100	–
goat	199	100	–
dog	199	100	–
cat	199	100	–
elephant	199	100	–
dolphin	199	100	–
megabat	199	100	–
microbat	199	99	R^170^ > K T^176^ > A
opossum 1	199	99.5	T^30^ > N
opossum 2	199	99	T^30^ > N V^128^ > M
Tasmanian devil	199	99.5	T^30^ > N
koala	199	99.5	T^30^ > N
human	199	100	–

## Discussion

The focus of this study was to characterize *Zmat2* genes in mammals by analyzing data available in genomic and gene expression repositories, and to place these findings in an evolutionary context. Prior to this and to our recent report (Baral K, Rotwein P: The story of ZMAT2: a highly conserved and understudied human gene, manuscript submitted), there had been no publications on *ZMAT2*/*Zmat2* genes from any species, despite the significance of the protein in the fundamentals of eukaryotic pre-RNA splicing [12, 14]. Our main observations here have included, first, demonstrating that 6-exon *Zmat2* is a single-copy gene in all mammals studied, except for opossum, in which a gene duplication event occurring after the divergence of monotremes from other marsupials ~ 80 Myr ago [15, 26] has led to paired tandem *Zmat2* genes (Fig. 4). Second, we have elucidated the presence of *Zmat2* pseudogenes in at least ten different mammalian species, have demonstrated that they are not transcribed in a context in which authentic *Zmat2* is expressed (Table 4, Figs. 5 and 6) and have shown that they appear to have arisen recently in these genomes (Fig. 5e); and third, we have found that the ZMAT2 protein is highly conserved among mammals (Table 5, Fig. 7). Importantly, our data demonstrate that a strategy involving the focused and complementary examination of genomic and gene expression databases can lead to new insights about mammalian biology and gene evolution, and illustrate how investigating unstudied genes can lead to the development of new experimentally-testable hypotheses.

### The *Zmat2* gene and pseudogenes in mammals

The data described and examined here define *Zmat2* as a 6-exon gene in 18 different mammalian species representing 9 orders (Tables 2, 3, Figs. 3, 4). They are thus very similar to their human and non-human primate orthologs in terms of both gene organization and the encoded ZMAT2 protein (Baral K, Rotwein P: The story of ZMAT2: a highly conserved and understudied human gene, manuscript submitted), supporting the idea that the protein plays a conserved and potentially essential role in pre-RNA splicing and possibly in keratinocyte differentiation (see below).

Pseudogenes have been described in both prokaryotes and eukaryotes [27], and are fairly common in the human and in other mammalian genomes [27]. Preliminary analysis of data generated by ENCODE, performed nearly a decade ago had suggested that there are more than 10,000 pseudogenes in the human genome, comprising ~ 0.7% of the DNA sequence [28]. Among these pseudogenes, ~ 77.5% were thought to represent processed mRNAs that had been retro-transposed as individual DNA copies into the genome, and the other ~ 22.5% were thought to be the result of gene duplication events [28].

*Zmat*2 pseudogenes could be identified in about half of the mammals studied here, and in all evaluable cases were not expressed in organs or tissues in which authentic *Zmat2* could be detected readily (Fig. 6), thus marking them as ‘real’ pseudogenes, unlike what was shown recently for human *PP1R2*, in which at least four previously identified pseudogenes were transcribed, and thus should be considered as genes [25]. Remarkably, the number of *Zmat*2 pseudogenes varied among these species, ranging from 1 to 4 per mammal (Table 4, Fig. 5). In addition, although most *Zmat*2 pseudogenes contained components of all 6 *Zmat*2 exons, in the guinea pig genome, the pseudogene was composed of exons 4–6, and in platypus, copies of exon 2 and exon 3 were located on different genome segments (Table 4). In the rat genome, two partial copies of 461 nucleotides of *Zmat*2 exon 6 were found in different locations on the X chromosome, but these were not detected in any of the other mammals studied (Table 4). While the full-length pseudogenes seem likely to have arisen via retro-transposition of mRNAs as DNA copies back into the respective genomes [24], the origins of the partial *Zmat2* gene sequences in guinea pig, platypus, and rat are unclear. Since *Zmat*2 pseudogenes were not identified in half of the mammals analyzed here, and since phylogenetic analysis of the ‘full-length’ pseudogenes indicated that they were more similar to their paralogs than to any orthologous DNA sequences in other mammals (Fig. 5e), it seems likely that they arose independently in each species subsequent to its evolutionary divergence from its closest ancestors.

### ZMAT2 proteins

ZMAT2 proteins are remarkably similar to one another in the mammalian species examined in this manuscript. Only 7 amino acid substitution variants were detected, with none found in the zinc finger domain. Including human and non-human primate ZMAT2, the protein was identical in 18/27 different mammals, and at most a variant protein in a given species contained 2 amino acid differences (Table 5, Fig. 6, and (Baral K, Rotwein P: The story of ZMAT2: a highly conserved and understudied human gene, manuscript submitted)), although, in platypus, the NH_2_-terminus of the protein could not be characterized because of poor quality genomic DNA sequence. In addition, we had shown recently that ZMAT2 is remarkably non-polymorphic in humans (Baral K, Rotwein P: The story of ZMAT2: a highly conserved and understudied human gene, manuscript submitted), with only 41 different potential codon changes identified that predicted amino acid substitutions in over 280,000 alleles found in the gnomAD project [29], corresponding to just 0.014% of the alleles in the entire study population (Baral K, Rotwein P: The story of ZMAT2: a highly conserved and understudied human gene, manuscript submitted). This level of variation in the human population is 6–90-fold lower than detected previously for at least 19 other human genes [30–32]. Moreover, and unlike these other genes [30–32], no frame shift or splicing site alterations were found in human *ZMAT2* (Baral K, Rotwein P: The story of ZMAT2: a highly conserved and understudied human gene, manuscript submitted).

One possibility for the high level of conservation of ZMAT2 among mammals is that the protein plays a key role in pre-mRNA splicing. ZMAT2 and its yeast homolog Snu23 have been found in the spliceosome [12, 14], and based on structural data, the protein has been postulated to facilitate activation of the U6 snRNP at the 5′ splice site of the intron [14]. Human ZMAT2 also may have a more specialized function, as it was described as a negative regulator of human keratinocyte differentiation, potentially by blocking the splicing of selected primary gene transcripts [11]. Defining the specific functions of ZMAT2 by genetic or other approaches in one or more tractable organisms will be an important topic for future study.

## Conclusions

### Stitching together genes in pieces: improving the quality of genome resources

Publicly available genomic databases contain extensive information on genes from many species, and are valuable resources for the entire scientific community. Unfortunately, as shown here, the quality of available information in certain circumstances is very poor. In nearly two-thirds of the species studied here, the annotated *Zmat2* gene in Ensembl lacked either 5′ or 3 UTRs, or both (Table 1), and in some cases could be identified only by screening with exons from other mammals. These types of problems may be quite common, and appears to be the norm for *Zmat2* genes from other mammalian and non-mammalian vertebrates in Ensembl. Poor annotation also has been described for several other genes in multiple species [19, 33]. Ideally, the data quality in these genomic repositories should be nearly perfect, not only to enhance the opportunity for future discoveries, but also to minimize the propagation of false information in scientific publications.

### Final comments

It has been estimated that only a tiny fraction of the ~ 20,000 human protein coding genes has been evaluated [1–3]. In fact, a recent report has suggested that ~ 90% of human genes are understudied [3], including several, such as ZMAT2, that have been the main topic of only a single publication [11]. It is likely that these statistics are more dismal for genes in other mammals and in non-mammalian vertebrates, even including species such as mouse and zebrafish that are favorites of experimentalists [34, 35]. Certainly, a concerted effort to broaden discovery horizons by focusing on understudied and unstudied genes could lead to new insights of potentially high biological and biomedical significance.

## Methods

### Database searches and analyses

Genomic databases were accessed in the Ensembl Genome Browser (www.ensembl.org), initially by text search using ‘*Zmat2*’ as the query term (see Table 6 for species-specific data links). Additional searches were performed in Ensembl with BlastN under normal sensitivity (maximum e-value of 10; mis-match scores: 1,-3; gap penalties: opening 5, extension, 2; filtered low complexity regions, and repeat sequences masked) using as queries mouse *Zmat2* DNA fragments (*Mus musculus*, genome assembly GRCm38.p6). The following genome assemblies were examined: cat (*Felis catus*, Felis_catus_9.0), cow (*Bos taurus*, ARS-UCD1.2), dog (*Canis lupus familiaris*, CanFam3.1), dolphin (*Tursiops truncatus*, turTru1), elephant (*Loxodonta africana*, LoxAfr3.0), guinea pig (*Cavia porcellus*, cavpor3.0), goat (*Capra hircus*, ARS1), horse (*Equus caballus*, EquCab3.0), human (*Homo sapiens*, GRCh38.p12), koala (*Phascolarctos cinereus*, phaCin_unsw_v4.1), megabat (*Pteropus vampyrus*, pteVam1), microbat (*Myotis lucifugus*, Myoluc2.0), opossum (*Monodelphis domestica*, monDom5), pig (*Sus scrofa*, Sscrofa11.1), platypus (*Ornithorhynchus anatinus*, OANA5), rabbit (*Oryctolagus cuniculus*, OryCun2.0), rat (*Rattus norvegicus*, Rnor_6.0), sheep (*Ovis aries*, OAE_v3.1), and Tasmanian devil (*Sarcophilus harrisii*, Devil_ref v7.0). The highest scoring results in all cases mapped to the *Zmat2* gene, or in several species, to *Zmat2* and to *Zmat2* pseudogenes. As many searches were incomplete, additional queries were conducted using species-homologous *Zmat2* cDNAs when available to verify or extend initial results. The following *Zmat2* cDNAs were obtained from the NCBI nucleotide database: cow (accession number: NM_001080343), horse (JL616468), koala (XM_021005188), mouse (NM_025594), rat (NM_001135582), and sheep (GAAI01003789). The Uniprot browser (http://www.uniprot.org/) was the source for ZMAT2 protein sequences (Additional file 6: Table S3); in the absence of primary protein data, DNA sequences of *Zmat2* exons were translated using Serial Cloner 2.6 (see: http://serialbasics.free.fr/Serial_Cloner.html).

**Table 6:** Data links to *Zmat2* genes in the Ensembl Genome Browser

Species	Data link
mouse	https://useast.ensembl.org/Mus_musculus/Transcript/Summary?db=core;g=ENSMUSG00000001383;r=18:36793876-36799666;t=ENSMUST00000001419
rat	https://useast.ensembl.org/Rattus_norvegicus/Transcript/Summary?db=core;g=ENSRNOG00000016016;r=18:29639872-29644652;t=ENSRNOT00000021516
guinea pig	https://useast.ensembl.org/Cavia_porcellus/Transcript/Summary?db=core;g=ENSCPOG00000037729;r=DS562861.1:2993661-3003969;t=ENSCPOT00000040194
rabbit	https://useast.ensembl.org/Oryctolagus_cuniculus/Transcript/Summary?db=core;g=ENSOCUG00000004255;r=3:22819773-22827697;t=ENSOCUT00000004254
cow	https://useast.ensembl.org/Bos_taurus/Transcript/Summary?db=core;g=ENSBTAG00000005441;r=7:51825588-51830683;t=ENSBTAT00000007159
horse	https://useast.ensembl.org/Equus_caballus/Transcript/Summary?db=core;g=ENSECAG00000014412;r=14:35453525-35458514;t=ENSECAT00000015031
pig	https://useast.ensembl.org/Sus_scrofa/Gene/Summary?db=core;g=ENSSSCG00000029158;r=2:142411089-142419358;t=ENSSSCT00000027327
sheep	https://useast.ensembl.org/Ovis_aries/Transcript/Summary?db=core;g=ENSOARG00000018690;r=5:49209879-49214290;t=ENSOART00000020342
goat	https://useast.ensembl.org/Capra_hircus/Transcript/Summary?db=core;g=ENSCHIG00000014775;r=7:58669461-58673620;t=ENSCHIT00000021084
dog	https://useast.ensembl.org/Canis_familiaris/Transcript/Summary?db=core;g=ENSCAFG00000005907;r=2:35852109-35858065;t=ENSCAFT00000009526
cat	https://useast.ensembl.org/Felis_catus/Transcript/Summary?db=core;g=ENSFCAG00000001289;r=A1:118951931-118956529;t=ENSFCAT00000001289
elephant	https://useast.ensembl.org/Loxodonta_africana/Transcript/Summary?db=core;g=ENSLAFG00000018601;r=scaffold_1:58364445-58369056;t=ENSLAFT00000034808
dolphin	https://useast.ensembl.org/Tursiops_truncatus/Transcript/Summary?db=core;g=ENSTTRG00000001777;r=GeneScaffold_3060:26788-31759;t=ENSTTRT00000001775
microbat	https://useast.ensembl.org/Myotis_lucifugus/Transcript/Summary?db=core;g=ENSMLUG00000017525;r=GL429795:4878837-4885021;t=ENSMLUT00000017529
megabat	https://useast.ensembl.org/Pteropus_vampyrus/Transcript/Summary?db=core;g=ENSPVAG00000009717;r=GeneScaffold_2046:692976-697483;t=ENSPVAT00000009717
opossum	https://useast.ensembl.org/Monodelphis_domestica/Gene/Summary?db=core;g=ENSMODG00000043385;r=1:332335696-332341799;t=ENSMODT00000056614;tl=aFRvNQY0dmA0F2ZJ-5959822-761277754 (gene 1) https://useast.ensembl.org/Monodelphis_domestica/Gene/Summary?db=core;g=ENSMODG00000038324;r=1:332342683-332348732;t=ENSMODT00000088321;tl=aFRvNQY0dmA0F2ZJ-5959822-761277755 (gene 2)
Tas. devil	https://useast.ensembl.org/Sarcophilus_harrisii/Transcript/Summary?db=core;g=ENSSHAG00000016524;r=GL834595.1:2209920-2216119;t=ENSSHAT00000019614
koala	https://useast.ensembl.org/Phascolarctos_cinereus/Transcript/Summary?db=core;g=ENSPCIG00000021080;r=MSTS01000108.1:4479556-4485102;t=ENSPCIT00000034728

### Mapping the 5′ and 3′ ends of *Zmat2* genes

Inspection of *ZMAT2* and its proposed mRNAs in the Ensembl genome database revealed for most species either a lack of 5′ or 3′ UTRs for *Zmat2* mRNAs, or poorly-defined 5′ or 3′ UTRs. In a few cases, as in horse, koala and sheep, a cDNA in the NCBI nucleotide database could be used to extend the 3′ UTR. For all species for which they were available, RNA-sequencing libraries found in the NCBI SRA (www.ncbi.nlm.nih.gov/sra) were queried with multiple 60 base pair probes from genomic DNA corresponding to presumptive 5′ portions of exon 1, and from 3′ parts of exon 6, and read counts were analyzed. All queries used the Megablast option (optimized for highly similar sequences; maximum target sequences–10,000 (this parameter may be set from 50 to 20,000); expect threshold–10; word size–11; match/mismatch scores–2, − 3; gap costs–existence 5, extension 2; low-complexity regions filtered). The RNA-sequencing libraries are listed in Additional file 1: Table S1, and the probes in Additional file 2: Table S2.

### DNA and protein alignments and phylogenetic trees

Multiple sequence alignments were performed for *Zmat2* pseudogenes from different species. DNA sequences were uploaded into the command line of Clustalw2 (https://www.ebi.ac.uk/Tools/msa/clustalw2/) [36] in FASTA format. A similar approach was used with ZMAT2 proteins, except that amino acid sequences were uploaded into Clustalw2 in FASTA format. Output files were in GCG MSF (Genetics Computer Group multiple sequence file) format, and were used as input into a command line form of IQ-TREE (http://iqtree.cibiv.univie.ac.at/), a software tool that uses maximum likelihood to generate phylogenetic trees [37]. IQ-TREE combines phylogenetic and combinatorial optimization techniques into a fast and effective tree search algorithm. The input sequence was bootstrapped 1000 times to get the optimal tree. The output file (with an extension of ‘.*filetree’*) became input into iterative Tree of Life (iTOL; https://itol.embl.de/), to produce pictorial phylogenetic trees. Pairwise alignments comparing the two ZMAT2 proteins discovered in opossum, and comparing ZMAT2 proteins with predicted proteins from *Zmat2* pseudogenes were performed using Needle (EMBOSS; see https://www.ebi.ac.uk/Tools/psa/), which creates an optimal global alignment of two sequences using the Needleman-Wunsch algorithm [36].

### Mapping pseudogenes

Initial screening of several mammalian genomes revealed more than one group of DNA sequences with high levels of identity with different mouse *Zmat2* exons, using the same BlastN criteria outlined above. In addition, when conceptually translated, many of these sequences resemble all or parts of ZMAT2 proteins (see Table 4). To determine if these DNA sequences were pseudogenes or actual genes [25], expression of transcripts was assessed in each species in which RNA-sequencing libraries were available in parallel with authentic *Zmat2* (see Fig. 6).

## Supplementary information

**Additional file 1: Table S1.** RNA-sequencing libraries screened for gene expression.

**Additional file 2: Table S2.** Probes for screening RNA-sequencing libraries.

**Additional file 3.** Characterizing 5’ ends of mammalian Zmat2 genes by analysis of RNA-sequencing libraries. Mapping putative 5’ ends of mammalian Zmat2 genes by examination of gene expression data from species-specific RNA-sequencing libraries, with 60 base pair genomic segments a-c, a-d, or a-e as probes. A. Rat; B. Guinea pig; C. Rabbit; D. Cow; E. Horse; F. Pig; G. Sheep; H. Goat; I. Megabat; J. Dog K. Cat; L. Elephant; M. Dolphin; N. Tasmanian devil; O. Koala.

**Additional file 4.** Characterizing 3’ ends of mammalian Zmat2 genes by analysis of RNA-sequencing libraries. Mapping putative 3’ ends of mammalian Zmat2 genes by examination of gene expression data from species-specific RNA-sequencing libraries, with 60 base pair genomic segments a-d, a-e, a-f, or a-g as probes. A. Rat; B. Guinea pig; C. Rabbit; D. Cow; E. Horse; F. Pig; G. Sheep; H. Goat. A vertical arrow denotes the possible 3’ end of Zmat2 transcripts.

**Additional file 5.** Characterizing 3’ ends of mammalian Zmat2 genes by analysis of RNA-sequencing libraries. Mapping putative 3’ ends of mammalian Zmat2 genes by examination of gene expression data from species-specific RNA-sequencing libraries, with 60 base pair genomic segments a-d, a-e, or a-f as probes. A. Dog; B. Cat; C. Elephant; D. Dolphin; E. Megabat; F. Koala; G. Tasmanian devil. A vertical arrow denotes the possible 3’ end of Zmat2 transcripts, which could not be identified for dog or cat genes.

**Additional file 6: Table S3.** Mammalian ZMAT2 protein sequences from UniProt.

## Data Availability

See Table 6 for data links and see specific accession numbers in Methods section above.

## Abbreviations

NCBI: National Center for Biotechnology Information
SRA: Sequence Read Archive
UTR: untranslated region

## References

[1] Oprea TI, Bologa CG, Brunak S (2018). Unexplored therapeutic opportunities in the human genome. Nat Rev Drug Discov.

[2] Haynes WA, Tomczak A, Khatri P (2018). Gene annotation bias impedes biomedical research. Sci Rep.

[3] Stoeger T, Gerlach M, Morimoto RI, Nunes Amaral LA (2018). Large-scale investigation of the reasons why potentially important genes are ignored. PLoS Biol.

[4] Manolio TA, Fowler DM, Starita LM (2017). Bedside back to bench: building bridges between basic and clinical genomic research. Cell..

[5] Battle A, Brown CD, Engelhardt BE, Montgomery SB (2017). Genetic effects on gene expression across human tissues. Nature..

[6] Soumillon M, Cacchiarelli D, Semrau S, van Oudenaarden A, Mikkelsen TS. Characterization of directed differentiation by high-throughput single-cell RNA-Seq bioRxiv 2014;1:1–13.

[7] Vera M, Biswas J, Senecal A, Singer RH, Park HY (2016). Single-cell and single-molecule analysis of gene expression regulation. Annu Rev Genet.

[8] Katsanis N (2016). The continuum of causality in human genetic disorders. Genome Biol.

[9] Quintana-Murci L (2016). Understanding rare and common diseases in the context of human evolution. Genome Biol.

[10] Acuna-Hidalgo R, Veltman JA, Hoischen A (2016). New insights into the generation and role of de novo mutations in health and disease. Genome Biol.

[11] Tanis SEJ, Jansen PWTC, Zhou H (2018). Splicing and chromatin factors jointly regulate epidermal differentiation. Cell Rep.

[12] Plaschka C, Lin PC, Nagai K (2017). Structure of a pre-catalytic spliceosome. Nature..

[13] Papasaikas P, Valcarcel J (2016). The spliceosome: the ultimate RNA chaperone and sculptor. Trends Biochem Sci.

[14] Bertram K, Agafonov DE, Dybkov O (2017). Cryo-EM structure of a pre-catalytic human spliceosome primed for activation. Cell.

[15] Bininda-Emonds OR, Cardillo M, Jones KE (2007). The delayed rise of present-day mammals. Nature..

[16] Nikolaev SI, Montoya-Burgos JI, Popadin K, Parand L, Margulies EH, Antonarakis SE (2007). Life-history traits drive the evolutionary rates of mammalian coding and noncoding genomic elements. Proc Natl Acad Sci U S A.

[17] Asher RJ, Bennett N, Lehmann T (2009). The new framework for understanding placental mammal evolution. Bioessays..

[18] Liu L, Zhang J, Rheindt FE (2017). Genomic evidence reveals a radiation of placental mammals uninterrupted by the KPg boundary. Proc Natl Acad Sci U S A.

[19] Rotwein P (2018). The insulin-like growth factor 2 gene and locus in nonmammalian vertebrates: organizational simplicity with duplication but limited divergence in fish. J Biol Chem.

[20] Rotwein P (2019). Quantifying promoter-specific insulin-like growth factor 1 gene expression by interrogating public databases. Phys Rep.

[21] Albright SR, Tjian R (2000). TAFs revisited: more data reveal new twists and confirm old ideas. Gene..

[22] Vo Ngoc L, Wang YL, Kassavetis GA, Kadonaga JT (2017). The punctilious RNA polymerase II core promoter. Genes Dev.

[23] Proudfoot NJ (2011). Ending the message: poly(a) signals then and now. Genes Dev.

[24] Weiner AM, Deininger PL, Efstratiadis A (1986). Nonviral retroposons: genes, pseudogenes, and transposable elements generated by the reverse flow of genetic information. Annu Rev Biochem.

[25] Korrodi-Gregorio L, Abrantes J, Muller T (2013). Not so pseudo: the evolutionary history of protein phosphatase 1 regulatory subunit 2 and related pseudogenes. BMC Evol Biol.

[26] Mitchell KJ, Pratt RC, Watson LN (2014). Molecular phylogeny, biogeography, and habitat preference evolution of marsupials. Mol Biol Evol.

[27] Mighell AJ, Smith NR, Robinson PA, Markham AF (2000). Vertebrate pseudogenes. FEBS Lett.

[28] Alexander RP, Fang G, Rozowsky J, Snyder M, Gerstein MB (2010). Annotating non-coding regions of the genome. Nat Rev Genet.

[29] Karczewski KJ, Laurent C Francioli, Grace Tiao, Beryl B Cummings, Jessica Alföldi, Qingbo Wang, Ryan L Collins, Kristen M Laricchia, Andrea Ganna, Daniel P Birnbaum, Laura D Gauthier, Harrison Brand, Matthew Solomonson, Nicholas A Watts, Daniel Rhodes, Moriel Singer-Berk, Eleanor G Seaby, Jack A Kosmicki, Raymond K Walters, Katherine Tashman, Yossi Farjoun, Eric Banks, Timothy Poterba, Arcturus Wang, Cotton Seed, Nicola Whiffin, Jessica X Chong, Kaitlin E Samocha, Emma Pierce-Hoffman, Zachary Zappala, Anne H O’Donnell-Luria, Eric Vallabh Minikel, Ben Weisburd, Monkol Lek, James S Ware, Christopher Vittal, Irina M Armean, Louis Bergelson, Kristian Cibulskis, Kristen M Connolly, Miguel Covarrubias, Stacey Donnelly, Steven Ferriera, Stacey Gabriel, Jeff Gentry, Namrata Gupta, Thibault Jeandet, Diane Kaplan, Christopher Llanwarne, Ruchi Munshi, Sam Novod, Nikelle Petrillo, David Roazen, Valentin Ruano-Rubio, Andrea Saltzman, Molly Schleicher, Jose Soto, Kathleen Tibbetts, Charlotte Tolonen, Gordon Wade, Michael E Talkowski, The Genome Aggregation Database Consortium, Benjamin M Neale, Mark J Daly, Daniel G MacArthur. Variation across 141,456 human exomes and genomes reveals the spectrum of loss-of-function intolerance across human protein-coding genes. bioRxiv. 2019;10.1101/531210

[30] Rotwein P (2017). Variation in Akt protein kinases in human populations. Am J Phys Regul Integr Comp Phys.

[31] Rotwein P (2017). Large-scale analysis of variation in the insulin-like growth factor family in humans reveals rare disease links and common polymorphisms. J Biol Chem.

[32] Rotwein P (2019). Variation in the repulsive guidance molecule family in human populations. Phys Rep.

[33] Rotwein P (2017). Diversification of the insulin-like growth factor 1 gene in mammals. PLoS One.

[34] White BH (2016). What genetic model organisms offer the study of behavior and neural circuits. J Neurogenet.

[35] Kawakami K, Largaespada DA, Ivics Z (2017). Transposons as tools for functional genomics in vertebrate m odels. Trends Genet.

[36] Madeira F, Park YM, Lee J (2019). The EMBL-EBI search and sequence analysis tools APIs in 2019. Nucleic Acids Res.

[37] Trifinopoulos J, Nguyen LT, von Haeseler A, Minh BQ (2016). W-IQ-TREE: a fast online phylogenetic tool for maximum likelihood analysis. Nucleic Acids Res.

