# Supplementary material for: Zmat2 in mammals: conservation and diversification among genes and Pseudogenes
Source: BMC Genomics. 2020 Jan 31;21:113. doi: 10.1186/s12864-020-6506-3 (PMC6995233; doi:10.1186/s12864-020-6506-3)
Supplement: Supplementary file 1 — Additional file 1: Table S1. RNA-sequencing libraries screened for gene expression. [file 12864_2020_6506_MOESM1_ESM.docx]

Additional Table 1: RNA-sequencing libraries screened for gene expression

| **Species** | **Tissue** | **Experiment** | **Platform** | **Layout** | **Reads sequenced**  **(x 10^6^)** |
| --- | --- | --- | --- | --- | --- |
| mouse | liver | SRX116916 | Illumina | single | 15.2 |
| mouse | keratinocyte | SRX5623906 | BGISEQ | single | 24.1 |
| rat | liver | SRX5590888 | Illumina | paired | 28.6 |
| guinea pig | liver | ERX1403335 | Illumina | paired | 50.6 |
| guinea pig | skin | SRX2789611 | Illumina | single | 30.5 |
| rabbit | skin | SRX2973129 | Illumina | paired | 88.0 |
| cow | liver | SRX196348 | Illumina | paired | 103.0 |
| horse | liver | ERX2600971 | Illumina | paired | 35.3 |
| pig | subcutaneous fat | ERX2259439 | Illumina | paired | 26.2 |
| sheep | liver | SRX4317440 | Illumina | single | 29.8 |
| goat | liver | SRX1538141 | Illumina | paired | 22.5 |
| dog | cardiac fat pad | SRX393125 | Illumina | single | 50.0 |
| cat | skin | SRX1625943 | Illumina | paired | 77.8 |
| elephant | fibroblasts | SRX3407592 | Illumina | paired | 105.2 |
| dolphin | epidermal cell line | SRX2833154 | Illumina | single | 37.5 |
| megabat | unspecified | ERX2072156 | Ion torrent | single | 38.8 |
| opossum | limbs | SRX3040092 | Illumina | single | 68.4 |
| Tas. devil | fibroblasts | SRX3474992 | Illumina | paired | 56.0 |
| koala | liver | SRX501262 | Illumina | paired | 113.4 |
