# Supplementary material for: Zmat2 in mammals: conservation and diversification among genes and Pseudogenes
Source: BMC Genomics. 2020 Jan 31;21:113. doi: 10.1186/s12864-020-6506-3 (PMC6995233; doi:10.1186/s12864-020-6506-3)
Supplement: Supplementary file 2 — Additional file 2: Table S2. Probes for screening RNA-sequencing libraries. [file 12864_2020_6506_MOESM2_ESM.docx]

Additional Table 2: Probes for screening RNA-sequencing libraries

| **Gene** | **Probe** |
| --- | --- |
| **rat** |  |
| *ZMAT2* ex 1-2 | TTCGCTGTGAAGATGGCGTCTGGCAGCGGGACAAAAAACTTGGACTTTCGCCGAAAGTGG |
| *ZMAT2* exon 3 | GAAAACCAGTGCAGCCAGTTAAGCGGGAGCTTCTACGGCACAGGGATTACAAGGTGGACT |
| *ZMAT2* ps exon 3 | GAAAACCAGCGCAGACAGTCTACTGGGAGCTTCTACCTCACGGGGATTGCAAGGTGGACT |
| **rabbit** |  |
| *ZMAT2* ex 1-2 | TTCGCTGTGAAGATGGCGTCGGGCAGCGGGACAAAAAACTTGGACTTTCGCCGAAAGTGG |
| *ZMAT2* exon 3 | CAGTGCAGCCAGTCAAGCGGGAACTTCTGCGACACAGAGACTACAAAGTGGACCTGGAAT |
| *ZMAT2* ps exon 3 | CAGTGCAGCCGGTCAAGCGGGAACTTCTGCGACACAGAGACTACAAAGTGGACCTGGAAT |
| **cow** |  |
| *ZMAT2* ex 1-2 | TTCGCAGTGAAGATGGCGTCAGGCAGTGGGACAAAAAACTTGGACTTTCGCCGAAAGTGG |
| **pig** |  |
| *ZMAT2* ex 1-2 | CGCTGTGAAGATGGCGTCGGGTAGCGGGGTCAAAAAACTTGGACTTTCGCCGAAAGTGGG |
| **sheep** |  |
| *ZMAT2* ex 1-2 | TCACTTCGCAGTGAAGATGGCGTCAGGCAGACAAAAAACTTGGACTTTCGCCGAAAGTGG |
| **goat** |  |
| *ZMAT2* ex 1-2 | TTCGCAGTGAAGATGGCGTCAGGCAGTGGGACAAAAAACTTGGACTTTCGCCGAAAGTGG |
| **dog** |  |
| *ZMAT2* ex 1-2 | TTCGCAGTGAAGATGGCGTCGGGCAGCGGGACAAAAAACTTGGACTTTCGCCGAAAGTGG |
| *ZMAT2* exon 3 | AAACCAGTACAGCCAGTCAAACGGGAGCTTCTCCGGCATAGGGACTATAAAGTGGACCTG |
| *ZMAT2* ps exon 3 | AAACCAGTACAGCCAGTCAAACGGGAGCTTCTCCGGCATAGAGACTATAAAGTGGACCTG |
| **cat** |  |
| *ZMAT2* ex 1-2 | TTCGCAGTGAAGATGGCGTCCGGCAGCGGGAAAAACTTGGACTTTCGCCGAAAGTGGGAC |
| **dolphin** |  |
| *ZMAT2* ex 1-2 | TTCGAAGTGAAGATGGCGTCGGGCAGTGGGACAAAAAACTTGGACTTTCGCCGAAAGTGG |
| *ZMAT2* exon 6 | GAGGAAAAGGCCAAGGCCTACAAGAAAGAGAAACAGAAGGAGAAGAAAAGGAGGGCTGAG |
| *ZMAT2* ps1 exon 3 | GAGGAAAAGGCTAAAGCCTACAAGAAAGAGAAACAGAAGGAGAAGAAAAGGAGGGCTGAG |
| *ZMAT2* exon 3 | AAACCAGTGCAGCCAGTCAAGCGAGAGCTTCTCCGGCATAGGGACTACAAGGTGGACCTG |
| *ZMAT2* ps2 exon 3 | AAACCAGTGCAGCCAGTCACGTGAGAGCTTCTCCGGCATCGGGACTCCAAGGTGGACCTG |
| *ZMAT2* ps3 exon 3 | AAACCAGTGCAGCCAGTCACGCAAGAGCTTCTCCAGCATCGGGACTCCAAGGTGGACCTG |
| *ZMAT2* ps4 exon 3 | AAACCAGTGCGGCCAGGGAAGCGAGAGCTTCTCCGGCATAGGGACTCCAAGGTGGACCTG |
| **guinea pig** |  |
| *ZMAT2* exon 6 | GAGGAGAAGGCTAAAGCATACAAGAAAGAGAAACAGAAGGAAAAGAAGAGGAGGGCCGAG |
| *ZMAT2* ps exon 6 | GAGGAGAAGGCTAAAGCATACAAGAAAAAGAAACAGAAGGCAAAGAAGAGGAGGGCCGAG |
| **megabat** |  |
| *ZMAT2* ex 1-2 | TTCGCGGTGAAGATGGCGTCGGGCAGCGGGACAAAAAACTTGGACTTTCGCCGAAAGTGG |
| *ZMAT2* exon 3 | AAACCAGTGCAACCAGTCAAGCGGGAGCTTCTCCGGCATAGGGACTACAAAGTGGACTTG |
| *ZMAT2* ps exon 3 | AAACCGGTGCACCCGGTCAAGTGGGAGCTTCTCTGGCATAGGGACTAGAAGGTGGACTTG |
| **opossum** |  |
| *ZMAT2-1* ex 1-2 | TCCACCGCGAAGATGGCGTCTGGCAGCGGGACTAAAAACCTGGACTTCCGCCGAAAGTGG |
| *ZMAT2-2* ex 1-2 | TCCACCGCGAAGATGGCGTCTGGTAGCGGGACTAAAAACCTGGACTTCCGCCGAAAGTGG |
| *ZMAT2-1* ex 6 | GAAGAGAAGGCCAAAGCCTACAAGAAGGAGAAACAGAAGGAGAAGAAACGAAGGGCAGAA |
| *ZMAT2-2* ex 6 | GAAGAGAAGGCCAAAGCCTACAAGAAGGAGAAACAGAAGGAAAAGAAACGAAGGGCAGAA |
| *ZMAT2* exon 3 | AAACCAGTGCAGCCTGTCAAGCGGGAGCTCTTGCGACATAGGGACTACAAAGTTGACTTG |
| *ZMAT2* ps exon 3 | ACACCAGTGAAGCCTGTCAAGCGGGAGCTCTTACGGCATAGGGATTACAAAGGTGATTTG |

*ps = pseudogene
