# Supplementary figures and images for: Zmat2 in mammals: conservation and diversification among genes and Pseudogenes
Source: BMC Genomics. 2020 Jan 31;21:113. doi: 10.1186/s12864-020-6506-3 (PMC6995233; doi:10.1186/s12864-020-6506-3)

Additional Fig. 1

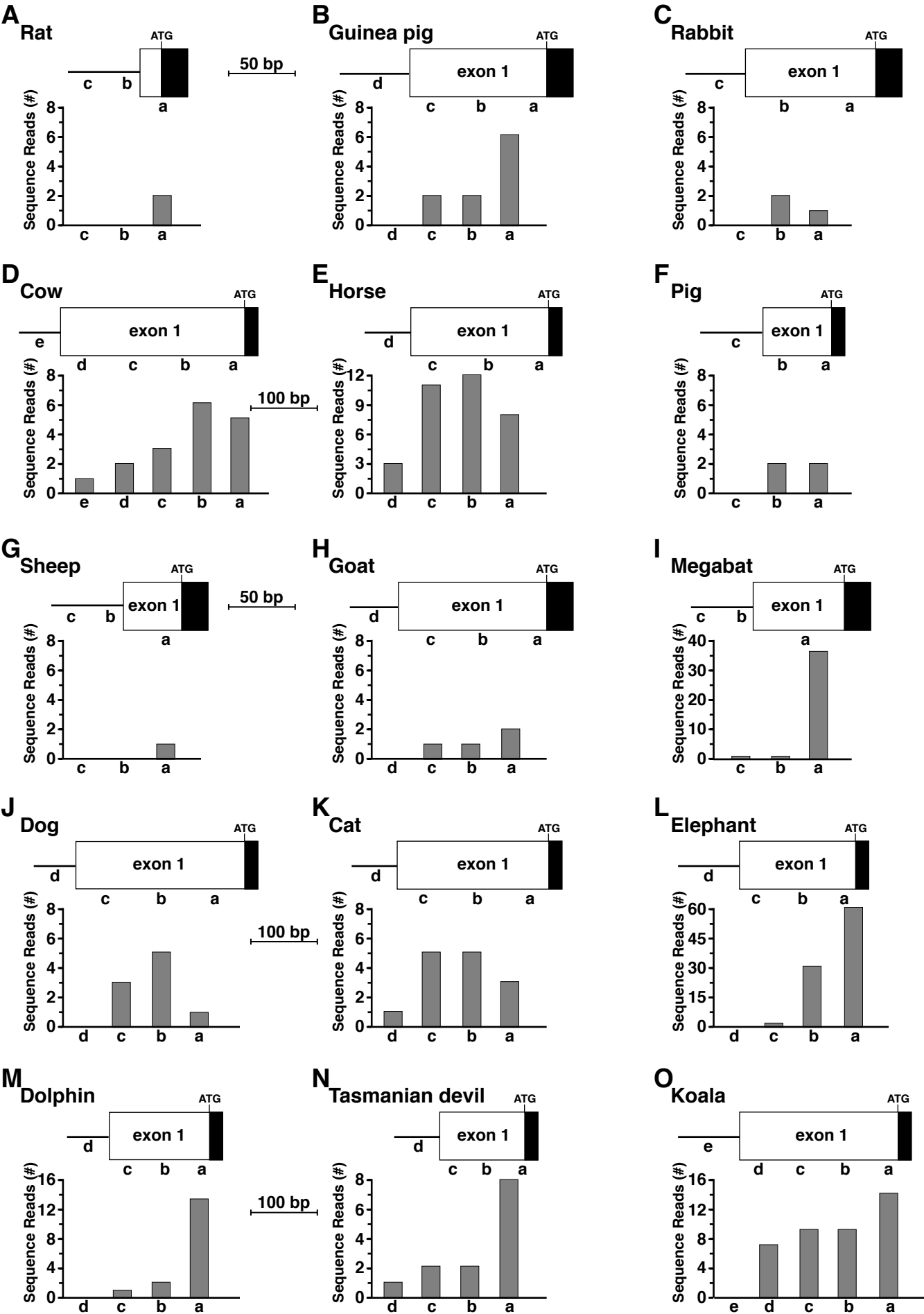

Supplement: Supplementary file 3 — Additional file 3. Characterizing 5’ ends of mammalian Zmat2 genes by analysis of RNA-sequencing libraries. Mapping putative 5’ ends of mammalian Zmat2 genes by examination of gene expression data from species-specific RNA-sequencing libraries, with 60 base pair genomic segments a-c, a-d, or a-e as probes. A. Rat; B. Guinea pig; C. Rabbit; D. Cow; E. Horse; F. Pig; G. Sheep; H. Goat; I. Megabat; J. Dog K. Cat; L. Elephant; M. Dolphin; N. Tasmanian devil; O. Koala. [file 12864_2020_6506_MOESM3_ESM.pdf]

Additional Fig. 2

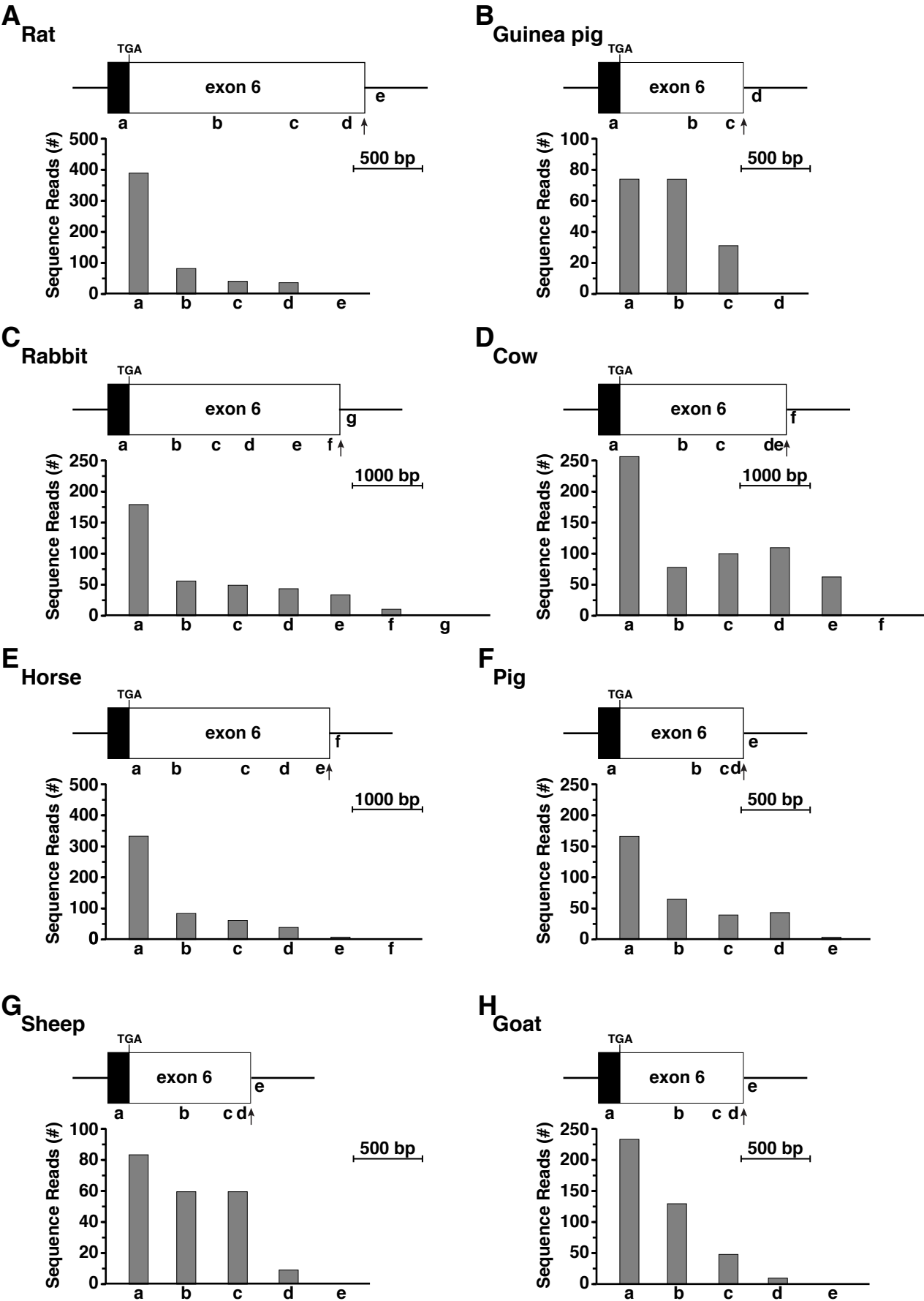

Supplement: Supplementary file 4 — Additional file 4. Characterizing 3’ ends of mammalian Zmat2 genes by analysis of RNA-sequencing libraries. Mapping putative 3’ ends of mammalian Zmat2 genes by examination of gene expression data from species-specific RNA-sequencing libraries, with 60 base pair genomic segments a-d, a-e, a-f, or a-g as probes. A. Rat; B. Guinea pig; C. Rabbit; D. Cow; E. Horse; F. Pig; G. Sheep; H. Goat. A vertical arrow denotes the possible 3’ end of Zmat2 transcripts. [file 12864_2020_6506_MOESM4_ESM.pdf]

Additional Fig. 3

**A**  
Dog

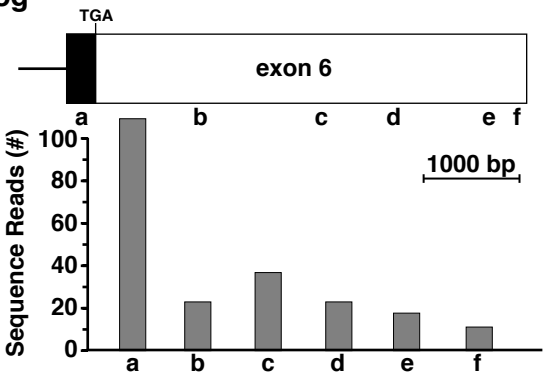

**B**  
Cat

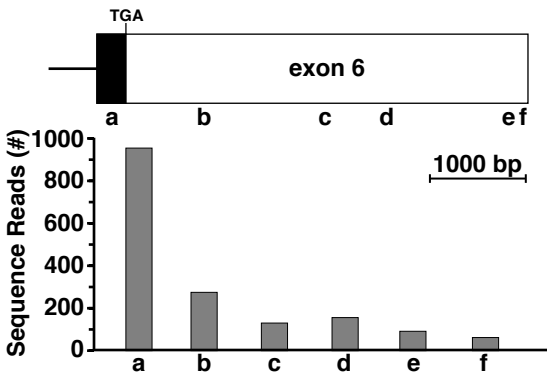

**C**  
Elephant

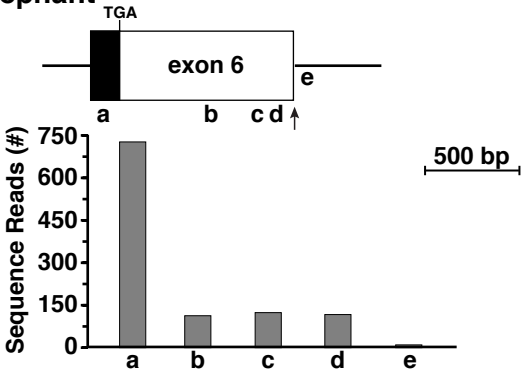

**D**  
Dolphin

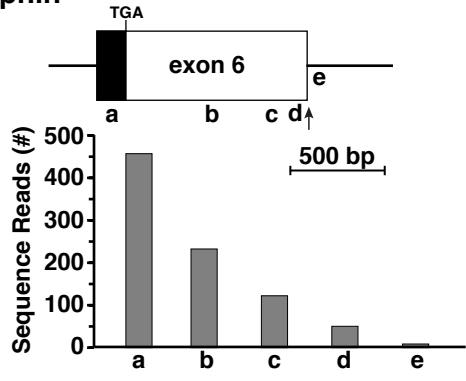

**E**  
Megabat

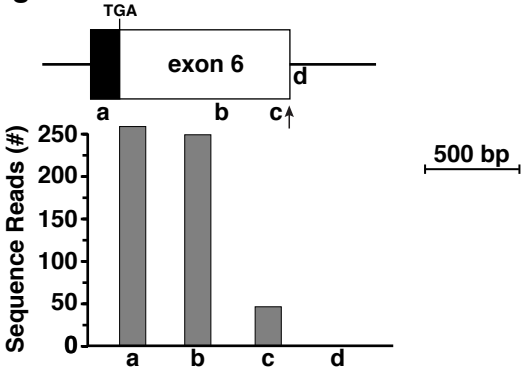

**F**  
Koala

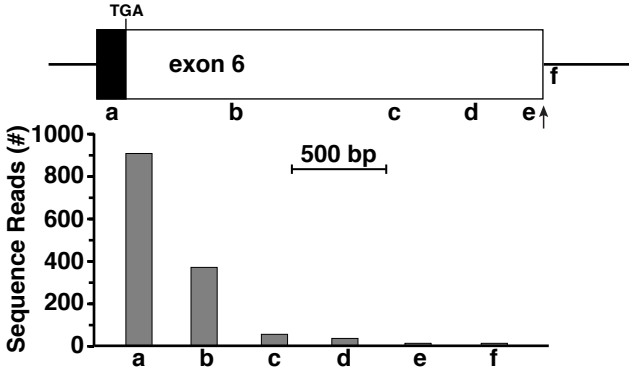

**G**  
Tasmanian devil

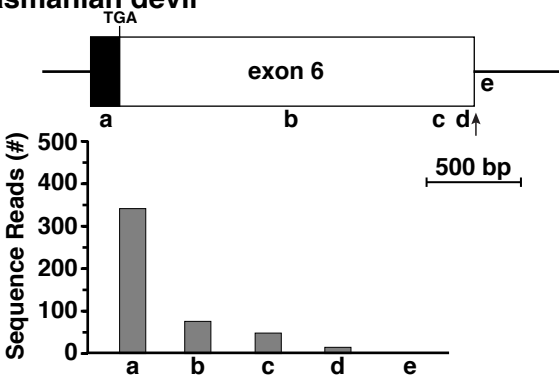

Supplement: Supplementary file 5 — Additional file 5. Characterizing 3’ ends of mammalian Zmat2 genes by analysis of RNA-sequencing libraries. Mapping putative 3’ ends of mammalian Zmat2 genes by examination of gene expression data from species-specific RNA-sequencing libraries, with 60 base pair genomic segments a-d, a-e, or a-f as probes. A. Dog; B. Cat; C. Elephant; D. Dolphin; E. Megabat; F. Koala; G. Tasmanian devil. A vertical arrow denotes the possible 3’ end of Zmat2 transcripts, which could not be identified for dog or cat genes. [file 12864_2020_6506_MOESM5_ESM.pdf]
