# Supplementary material for: Zmat2 in mammals: conservation and diversification among genes and Pseudogenes
Source: BMC Genomics. 2020 Jan 31;21:113. doi: 10.1186/s12864-020-6506-3 (PMC6995233; doi:10.1186/s12864-020-6506-3)
Supplement: Supplementary file 6 — Additional file 6: Table S3. Mammalian ZMAT2 protein sequences from UniProt. [file 12864_2020_6506_MOESM6_ESM.docx]

Additional Table 3: Mammalian ZMAT2 protein sequences from UniProt

| **Species** | **Entry number** |
| --- | --- |
| mouse | Q9CPW7 |
| rat | D4AD01 |
| guinea pig | A0A286XSH8 |
| rabbit | G1SLE4 |
| cow | Q0P584 |
| horse | F7CNZ5 |
| pig | I3LF90 |
| sheep | W5QBG7 |
| goat | A0A452EN96 |
| dog | E2QZ35 |
| cat | M3VWD5 |
| elephant | G3TL52 |
| dolphin | A0A2U4AGM6 |
| microbat | G1PX26 |
| megabat | No data |
| opossum | No data |
| Tas. devil | G3WVK2 |
| koala | No data |
